# Psychological impact of the covid-19 pandemic on the operating room paramedical staff

**DOI:** 10.1192/j.eurpsy.2022.366

**Published:** 2022-09-01

**Authors:** H. Hamada, M. Soussi, R. Chrigui, S. Guellim, M. Kahloul, W. Naija

**Affiliations:** Sahloul Academic Hospital, University of medicine, “Ibn Al Jazzar”, Sousse, Tunisia, Department Of Anesthesia And Intensive Care, Sousse, Tunisia

**Keywords:** COVID19, Anxiety, Depression, operating room

## Abstract

**Introduction:**

the recent covid19 pandemic is not devoid of psychological risks on paramedical staff. Among them, those who work in the operating theaters are exposed to such risks.

**Objectives:**

to determine the perceived stress level and the psychological impact of COVID-19 on paramedics in the operating room.

**Methods:**

This is an observational, descriptive and analytical study carried out in the operating rooms of Sahloul University Hospital during a 3 month period. The data collection tool was a self-administered questionnaire composed of 5 main parts (socio-demographic characteristics, occupational characteristics, exposure to COVID-19, the Perceived Stress Scale (PSS) and the Hospital Anxiety and depression scale (HADS)).

**Results:**

96 paramedical staff participated in our study. The average perceived stress score was significantly higher among anesthetists. 48% of participants had anxiety. Anesthetists had significantly higher anxiety scores (p = 0.001). 26.1% of participants had definite depression. Of those with definite depression, 35.3% were anesthetists (p = 0.028). Factors significantly associated with the occurrence of anxiety were: psychiatric history, increased workload, contact with a positive coronavirus patient in the operating room, and severe perceived stress. However, the factors significantly associated with the occurrence of depression were: initial training in the management of covid-19 patients, personal infection with SARS-COV2 and severe perceived stress.

**Conclusions:**

Covid-19 pandemic is causing significant symptoms of anxiety and depression among operation room staff. Primary and secondary prevention strategies must then be undertaken.

**Disclosure:**

No significant relationships.

